# A qualitative study of reinforcement workers’ perceptions and experiences of working in intensive care during the COVID-19 pandemic: A PsyCOVID-ICU substudy

**DOI:** 10.1371/journal.pone.0264287

**Published:** 2022-03-04

**Authors:** Florian Perraud, Fiona Ecarnot, Mélanie Loiseau, Alexandra Laurent, Alicia Fournier, Florent Lheureux, Christine Binquet, Jean-Philippe Rigaud, Nicolas Meunier-Beillard, Jean-Pierre Quenot

**Affiliations:** 1 Service d’Accueil des Urgences, University Hospital Dijon, and Université de Bourgogne Franche-Comté, Dijon, France; 2 Department of Cardiology, University Hospital Besançon, 25000 Besançon, France; 3 EA3920, University of Burgundy-Franche-Comté, 25000 Besançon, France; 4 Service de Médecine Légale, Cellule d’Urgence Médico-Psychologique de Bourgogne Franche-Comté, University Hospital Dijon, Dijon, France; 5 Laboratoire de Psychologie: Dynamiques Relationnelles Et Processus Identitaires (PsyDREPI), Université Bourgogne Franche-Comté, Dijon, France; 6 Department of Anaesthesiology and Critical Care Medicine, University Hospital Dijon, Dijon, France; 7 Laboratoire de Psychologie, University of Burgundy-Franche-Comté, 25000 Besançon, France; 8 Inserm CIC1432, module Épidémiologie Clinique (CIC-EC)- CHU Dijon-Bourgogne, UFR des Sciences de Santé, Dijon, France; 9 Service de Médecine Intensive-Réanimation, Hospital Centre of Dieppe, Dieppe, France; 10 Espace de Réflexion Éthique de Normandie, Université de Caen, Caen, France; 11 CIC 1432, Clinical Epidemiology, University of Burgundy, Dijon, France; 12 Direction de la Recherche Clinique et de l’Innovation, University Hospital Dijon, Dijon, France; 13 Service de Médecine Intensive-Réanimation, University Hospital Dijon, Dijon, France; 14 Equipe Lipness, centre de recherche INSERM UMR1231 et LabEx LipSTIC, université de Bourgogne-Franche Comté, Dijon, France; 15 Espace de Réflexion Éthique Bourgogne Franche-Comté (EREBFC), Dijon, France; Himachal Pradesh Medical College: Indira Gandhi Medical College, INDIA

## Abstract

**Purpose:**

During the COVID pandemic, many hospitals had to mobilize reinforcement healthcare workers, especially in intensive care (ICUs). We investigated the perceptions and experiences of reinforcement workers deployed to ICUs, and the impact of deployment on their personal and professional lives.

**Methods:**

For this qualitative study, a random sample of 30 reinforcement workers was drawn from 4 centres participating in the larger PsyCOVID-ICU study. Individual semi-structured interviews were held, recorded, transcribed and analyzed by thematic analysis.

**Results:**

Thirty interviews were performed from April to May 2021 (22 nurses, 2 anesthesiology nurses, 6 nurses’ aides). Average age was 36.8±9.5 years; 7 participants had no ICU experience. Four major themes emerged, namely: (1) Difficulties with integration, especially for those with no ICU experience; (2) lack of training; (3) difficulties with management, notably a feeling of insufficient communication; (4) Mental distress relating to the unusual work and fear of contaminating their entourage.

**Conclusion:**

Healthcare workers deployed as reinforcements to ICUs at the height of the pandemic had a unique experience of the crisis, and identified important gaps in organisation and preparation. They also suffered from a marked lack of training, given the stakes in the management of critically ill patients in the ICU.

## Introduction

The COVID-19 pandemic has caused unprecedented disruption to healthcare delivery worldwide. Many hospitals rapidly became swamped, as was the case in France at the various peaks of the epidemic [1]. Capacity (in terms of number of beds) quickly had to be scaled up, with the creation of extra critical care beds, requiring a corresponding increase in qualified staff [1,2]. Massive reinforcement of the intensive care unit (ICU) workforce was achieved thanks to a complex reorganization process covering various domains of the hospital activity. Firstly, cancellation of surgical activity enabled redeployment of staff from the operating theatres, with 70% of surgical interventions descheduled according to some estimates [3]. Similarly, other wards and technical laboratories with reduced activity were able to redeploy staff to work in ICUs. However, the task of training these redeployed healthcare workers (HCWs), also called “reinforcement workers” [4], even if it only meant brushing up their skills, fell on the permanent ICU staff. Several authors have described the impact in terms of mental health of this sudden and sometimes unexpected influx of support staff in the ICU. In a study of 69 HCWs working in a 16-bed neurological ICU, Altmayer et al reported that regular ICU staff were at greater risk of developing psychological disorders compared with the reinforcement workers, with higher levels of depressive symptoms [4]. The authors explained this by the fact that regular ICU staff had to both deal with the first wave of the pandemic and train the reinforcement, with greater responsibility for any potential errors committed by the support staff, thus profoundly disturbing the usual working conditions. Conversely, in the PsyCOVID-ICU study [5], involving 77 hospitals throughout France, our group previously showed that support staff redeployed to the ICU were at higher risk of psychological distress as compared to permanent ICU staff.

To complement the quantitative findings obtained with questionnaires evaluating psychological distress among HCWs during this uniquely challenging period, we felt it was important to explore the perceptions of reinforcement staff who were redeployed to the ICU during the pandemic. Therefore, by means of semi-structured interviews performed with support staff who participated in the PsyCOVID-ICU study, we investigated their perceptions and experiences of the different COVID waves, and the impact on their personal and professional lives.

## Methods

This qualitative study was performed as part of the larger PsyCOVID-ICU study, whose methods and results have previously been reported elsewhere [5]. In brief, the PsyCOVID-ICU study was a large, prospective study performed in 77 hospitals throughout France from April 22, 2020 to July 13, 2021, in which 5 successive assessments were performed at regular intervals. Only the findings relating to psychological distress at the first assessment timepoint have been reported to date [5].

The PsyCOVID-ICU study received approval for all participating centres from the Ethics Committee of the French Intensive Care Society (N°20–33), for both the quantitative and qualitative parts. Consent to participate in the main PsyCOVID study was implied by the fact that all participants voluntarily connected to the study website and completed the forms. Participants also agreed to participation at the beginning of the survey before proceeding with completion of the forms, and provided specific consent to be contacted for a qualitative interview to explore their responses in greater detail. During the interviews for the present study, participants were informed that the interviews would be recorded and consented to this. They were also informed and consented to the fact that illustrative quotes might be used for the purposes of publication (after translation).

For the present qualitative study, each of the four participating centres (Dijon, Chalon/Saône, Auxerre, Trevenans) sent us the full list of all the reinforcement workers in their centre. This amounted to a total of 105 healthcare workers (HCWs) who worked in the intensive care unit (ICU) as support staff during either of the two waves of 2020 (March to May 2020 (wave 1) or September to November 2020 (wave 2)) in any of the four participating centres. These 105 HCWs were contacted by email by 3 researchers from the Psy-COVID-Qualitative team (FP, JPQ, NMB) to organize an individual semi-structured interview at a time convenient for the interviewee. Reminders were sent to non-responders.

We developed an interview guide focused on 3 mains topics: (1) What is your experience and your perception of critical care, in general? (2) Can you describe your temporary experience of working in the ICU? (3) In your opinion, how could we improve the integration of support staff in the ICU? The interview guide was developed by our research team (FP, NMB, FE, JPQ) in collaboration with the caregiving teams on the ground (physicians, paramedical staff and nursing management from the four participating centres), and based on a review of the relevant literature. The interview guide was pilot tested with 3 nurses from our Department; the findings from their interviews were not included in the analysis. The pilot interviews did not give rise to any major changes in the interview guide.

Due to the ongoing pandemic during the study period, all interviews were performed by telephone by FP. Interviews were performed until data saturation was reached. Interviews were recorded and fully transcribed (in French) for later analysis. Data were encoded to guarantee the anonymity of the participants. No software was used to assist with data management.

The transcripts of all interviews were analyzed using thematic analysis, as previously described [6–8]. In thematic analysis, the verbatim are first coded, then codes are regrouped into major and minor themes [8]. Major themes are significant points that are mentioned spontaneously and well developed by all participants in a cross-sectional manner. Minor themes are secondary points that are less well developed by some, but not all participants. The first level of analysis (familiarization with the data, generating initial codes) was performed individually by each researcher in the team, then meetings were held to harmonize and decide on the major and secondary themes to be retained, and their regrouping into subject categories (defining and naming the definitive themes). The analysis was validated by 4 authors (FP (reference author, male), NMB (male, sociologist, PhD), FE (female, clinical researcher, PhD), JPQ (male, critical care physician, MD, PhD). The final report was written by the same 4 authors, and approved by all. Differences in interpretation were resolved by discussion and consensus. Results were not returned to the participants. Translation was performed after the results were finalized. Participants were informed that citations from their discourse may be used (translated into English) to illustrate the results of the study in a scientific publication, and all participants consented to this.

## Results

A total of 105 reinforcement workers were contacted, of whom 42 (41%) agreed to participate in the interview. A total of 30 interviews were performed with reinforcement workers from April to May 2021, at which point saturation was reached in the data, and thus, no further interviews were performed. There were 22 nurses, 2 anaesthesiology nurses and 6 nurses’ aides. The average age of participants was 36.8±9.5 years. Seven participants had no experience of working in the ICU, while the other 23 had previously worked in an acute care or ICU environment. The average duration of the interviews was 23±5 minutes.

Four major themes emerged from the interviews, namely: (1) Difficulties with integration, especially for those who had no experience of ICU care; (2) Lack of training in intensive care; (3) Difficulties with the management team, notably a feeling of insufficient communication; (4) Mental distress related to the unusual work and the fear of contaminating their entourage.

**Fig 1:**
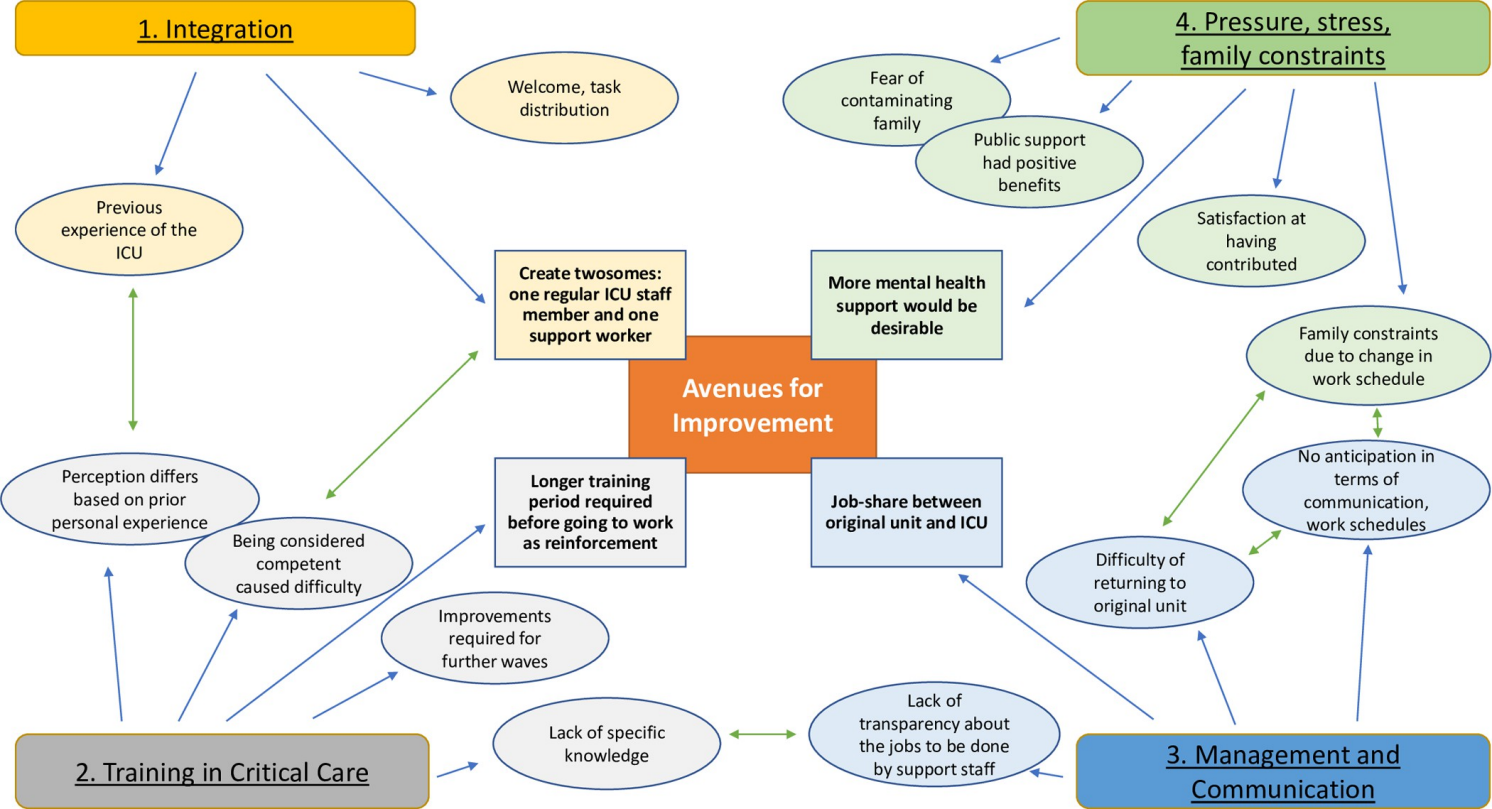
Conceptual framework summarizing the main themes.

Illustrative quotes are given in italics for each point. A conceptual framework summarizing the main themes is illustrated in Fig 1.

### 1/ Difficulties with integration, especially for those who had no experience of ICU care

One third of the participants reported that they had difficulties with the work organisation and task distribution when they first arrived in the ICU, with the feeling that their arrival was unexpected and not planned for. This feeling was unique to the first wave of the epidemic.

*“We arrived in the ICU, and no-one in the regular ICU team was expecting us. Nothing had been planned for how we would be assigned, or who’d be in charge of us. So I just hooked up with an ICU nurse and followed her around” (Registered Nurse (RN), 45 years old).*

Conversely, having some experience of the ICU or knowing the teams with whom they would be working was clearly beneficial in promoting earlier integration. It also made it easier to solve any problems (e.g. not knowing where things were stored, doubts about the use of certain drugs, or need for assistance in performing certain technical manoeuvers specific to intensive care).

*“I knew the unit and the teams working there, which made it much easier. They gave me a warm welcome and they were all very nice” (RN, 52 years old)*

All the participants noted that although the nursing managers tried their best to associate each reinforcement worker with a regular ICU staff member, it wasn’t always logistically possible.

*“They tried to have us work in nurse-and-aide twosomes with one regular, and one new person, but a lot of the time, there weren’t enough regular workers to avoid having two new people working together, and that created some problems” (RN, 45 years old)*

This point deserves to be underlined, since many of the reinforcement staff were new to intensive care, and mentoring by experienced staff from the ICU enabled some support staff to avoid making errors. Mentoring and supervision also limited the uneasiness associated with sudden immersion in a new and technical discipline in the middle of a crisis.

### 2/ Training in intensive care

The training received by the support staff was largely considered to be insufficient, both in terms of theoretical and practical teaching, especially during the first COVID wave.

*“I had some training with a resident, then I followed a nurse around the unit for a day. Then the next day, I was on my own with two patients to care for” (RN, 36 years old)*

The situations in this regard varied widely, due to the different levels of experience in the ICU, or the lack of knowledge of intensive care medicine.

*“I wasn’t mentored, but I didn’t really need to be. My work experience in the ICU was recent enough for me to be operational immediately” (student nurse, 34 years old)*

However, some reinforcement workers whose experience of ICU dated from a long time back, were uneasy with being considered as “immediately operational”, and would have appreciated some time to freshen their skills, or be trained in certain technical manoeuvers (e.g. aspiration via the endotracheal tube, dialysis, use of respirators etc).

*“I was mentored by a regular nurse’s aide who showed me lots of things. The reverse side of that coin is that she kept telling me I already knew because I’d worked in the ICU before. But I didn’t necessarily know–it’s been 10 years since I worked in the ICU, and I’ve lost the reflexes” (Nurse’s aide, 47 years old)*

Conversely, before the second wave of the epidemic, basic training was implemented. In one centre, for example, there was a 3-day training course given by ICU nurses and physicians including theoretical learning and practical simulation exercises (simulating of cardio-respiratory resuscitation, and presentation of common ICU equipment such as central venous lines or arterial catheters).

*“The training course concentrated a bit too much on theoretical knowledge, it was interesting but too detailed and not adapted to young nurses. The practical workshops with the manikins were good but we didn’t have enough time and it was aimed at a really wide audience” (RN, 45 years old)*

The participants were unanimous in stating that more training is necessary. A visit on site in the destination ICU, and a brief presentation of the organisation of the unit would also be helpful, to facilitate the integration into the regular teams, and to help make reinforcement staff operational more quickly, by giving them an idea of the environment and the culture of the ICU.

*“I would’ve liked to have a visit of the unit at the start, and for someone to show me where things were stocked, and the logistics. One day of immersion isn’t enough, you’d need at least a week” (RN, 45 years old)*

The choice of the mentors from among the regular staff could be made in advance, so that they could prepare the job in advance, and make an inventory of the minimum set of knowledge and/or competences to be transferred.

*“Just because you have experience and you work well doesn’t mean that you can train someone well. Not everyone is a good teacher, or wants to be. It would be good to have a team of volunteer mentors” (RN, 45 years old).*

Finally, some reinforcement staff felt that there was a lack of transparency at the initial presentation of the tasks to be done (e.g. the nursing manager assuring the support staff that they would not have to manage dialysis patients, but this was not respected). This compounded the support workers’ perception of being in difficulty performing jobs that were new to them. Communication with the nursing management and the hospital management emerged as a crucial actionable point with a view to improving the well-being at work of reinforcement staff.

### 3/ Difficulties with the management team, notably a feeling of insufficient communication

This problem was reported by the participants and across the successive waves of the pandemic. It highlights the lack of anticipation on the part of the management regarding where the reinforcements would be deployed, and how the work schedules would be organized, and this had repercussions on the personal lives of the reinforcements.

*“I only got my time schedule at the last minute. I felt like I was a pawn being pushed around, and nobody was worried about what I wanted, on the pretext that I was there to help out” (RN, 32 years old)*

There was a reported lack of transparency regarding the work that the reinforcements would have to do, with the result that they sometimes had to take on difficult tasks that they weren’t prepared for.

*“At the beginning, we were just meant to help out with tidying away the equipment, we weren’t supposed to be caring for the patients. Then very soon, they asked me to take over the care of a patient, then to do the dialysis. I couldn’t manage it all, and I felt like I was a burden on my colleagues” (RN, 53 years old).*

Several participants reported difficulties when they returned to their original unit, either because there had not been any anticipation in the work schedules of their return, or because their original job had changed in the meantime.

*“There was no anticipation of my return to my original unit. They asked me to take up a mobile position in the replacement pool, then a full-time position as a night nurse, but I worked days before, on a 4-day week” (RN, 32 years old)*

Proposals for shared activities between the original unit and the ICU would be appreciated to solve these problems. This solution might also help to mitigate the impact of the changing work schedules on the family life of reinforcement workers.

### 4/ Mental distress related to the unusual work and fear of contaminating entourage

The change in working hours (moving from seven and a half hour shifts to 12 hour shifts) led to significant fatigue and additional family-related constraints for the participants of this study.

*“I was really tired. I wasn’t used to working 12 hour shifts, I’d forgotten that it complete changes your everyday life” (RN, 53 years old)*

The first wave also generated a lot of anxiety for reinforcement personnel. On top of a heavy workload and equipment shortages (amplified by the media), they were also afraid that they would contaminate their families.

*“I wasn’t afraid of getting COVID myself but I felt that I was a danger to those around me. I was a danger to my family, putting them at risk because I might bring the virus home from work” (RN, 36 years old)*

Nevertheless, the outpouring of generosity among the population during the first wave of the epidemic (applause in the streets in the evening, gifts of food and equipment, pictures drawn by children etc), was a source of comfort to them, although looking back now, it didn’t last very long.

*“The generosity shown by the population was good for morale at the beginning, although we quickly faded back to just being ordinary healthcare workers” (RN, 39 years old)*

Conversely, despite the uncertain environment and the stressful work, numerous participants reported that they were satisfied, even proud to have participated in managing the crisis.

*“It stressed me a lot that I didn’t know how to do things, or that I forgot important things, or that I was burden on my colleagues. But despite all that, I’m glad I was able to contribute” (Nurse’s aide, 22 years old)*

Finally, considering the pressure generated by the jobs to be done, and the unusual working conditions, better communication about the possibility of psychological support appears to be desirable.

*“I didn’t know of any support services put in place, but it would’ve been good, because everybody was a bit vulnerable during that period” (Nurse’s aide, 47 years old)*

*“The psychologist used to come round in the morning before the handover, but after working all night, the only thing on your mind was getting home to bed” (RN, 23 years old).*

## Discussion

There are few data in the literature from qualitative studies among support workers redeployed to reinforce healthcare teams in ICUs during the COVID-19 crisis. As underlined in our study and others, there has been a substantial mental health impact of the crisis, both for regular staff working permanently in the ICU [4], who have been shown to have a non-negligible suicide risk [9], and for supporting staff working a short stint to reinforce the ICU workforce [5,10].

Our study brought forth four key issues for reinforcement workers. First, difficulties with integration, especially for those who had no experience of critical care; second, insufficient theoretical and practical training in critical care; third, difficulties with supervisors and hierarchy, who were no doubt overwhelmed by the difficulties of managing human resources, and a lack of communication from the hospital; and finally, psychological difficulties stemming from the atypical work, and a fear of contaminating their family with the virus. These points provide insights into possible avenues for improving the integration of reinforcement workers deployed to the ICU in times of crisis.

The integration of reinforcement workers can be difficult when the regular staff are used to working together in critical care, and also, when there may already be a certain level of psychological suffering present among the regular ICU workers [11,12]. Indeed, ICU staff have had to adapt their management of COVID-19 patients frequently (especially during the first wave). For example, recommendations for the use of personal protective equipment changed according to availability of the material; management practices changed in terms of respiratory support, with a scarcity of respirators and equipment; drugs were sometimes in short supply or totally out of stock; communication practices had to be modified when family visits were forbidden due to lockdown measures. Furthermore, ethical issues were also foremost, with the need for prioritisation of access to ICU due to a shortage of beds [2,13]. In a systematic literature review, Turner et al described primary qualitative studies depicting the experiences and perceptions of organisations and actors at multiple levels of health systems internationally in responding to COVID-19 [14]. They identified programmes to provide psychological support and improve leadership, as well as ideas for the development of communication tools, such as telemedicine. Some of these proposals would likely be relatively simple to implement to meet the various needs encountered by the participants of our study, both in terms of management practices, and communication with their hierarchy.

Regarding training, supervision and accompaniment during their time in the ICU, the reinforcement workers who participated in this study acknowledged that intensive care is very specific. There is a need to handle the emergency context, with a high density of care procedures requiring scrupulous hand hygiene (e.g. an average of 158 hand hygiene opportunities per patient over a 12-hour in the ICU, compared to 28 in conventional hospital wards [15,16]); mobilisation of technical, pathophysiological and pharmacological knowledge; understanding of a wide spectrum of diseases and life-support options; mastery of a complex environment with very critically ill patients with numerous comorbidities and a high risk of death [17–19]. With this in mind, the French Intensive Care Society (Société de Réanimation de Langue Française, SRLF) published guidelines in 2011 stipulating the minimum set of qualifications recommended for ICU nurses [20]. These recommendations are line with French legislation regarding the organisation of critical care. The wide panel of required skills necessitates several months, not to say years of training before becoming suitably proficient and capable of working in the ICU, which is also in line with recommendations from other countries [21–23].

One lesson learned from this crisis is that there was significant heterogeneity in the reinforcements deployed to ICUs, resulting in varying levels of competence. This in turn may have impacted on patient safety and quality of care [1]. In this context, experienced ICU nurses were responsible for mentoring reinforcement nurses, but it was a complicated task in view of the extremely heavy workload. In the literature, more innovative training approaches have been proposed [24] using simulated scenarios followed by debriefing sessions on attitude and discussion of practices. These authors reported that the prevalence of job strain at 6 months was significantly lower in the intervention group than in the control group (13% vs 67%, respectively, p<0.0001), and absenteeism during the 6-month follow-up was also significantly lower (1% vs 8%, intervention vs control groups, p = 0.03) [24].

At the height of the epidemic, the two French societies of intensive care (SRLF and SFAR) addressed the issue of training, and proposed a guide for reinforcement workers in the ICU in exceptional sanitary circumstances [25], with a view to guaranteeing that the increased bed capacity in ICUs was met with the necessary professional qualifications. Beyond ensuring high-quality training for caregivers, the aim of this document was to avoid massive de-scheduling of healthcare procedures, notably surgical procedures, during the various waves of the epidemic, due to the mobilization of the human resources available in operating theatres. Amongst other measures, this report proposes a digital passport (obtained after validating educational qualifications and immersion of several days in an ICU), which would guarantee the skill level of caregivers in a reserve pool available for deployment to hospitals at local and regional level.

The psychological stress caused by doing a job that is not one’s usual work, combined with the fear of catching or transmitting COVID, has been widely reported from China since the beginning of the pandemic [26–28].

This study has some limitations. First, the points addressed with the participants during the interviews may not have covered all the repercussions of the pandemic on their personal and/or professional lives. Second, only reinforcement workers who were deployed to centres participating in the PsyCOVID-ICU study and who agreed to participate were interviewed. Third, interviews were performed by telephone due to the lockdown conditions. This may have influenced how the participants responded, and non-verbal information may have been lost. Finally, all participants were French, and discussed the French experience of the pandemic. Therefore, results may not be generalizable to other settings.

## Conclusion

HCWs deployed as reinforcements to ICUs at the height of the pandemic had a unique experience of the crisis, and identified important gaps in organisation and preparation. They also suffered from a marked lack of training, given the stakes in the management of critically ill patients in the ICU. Similarly, the mental health of reinforcement workers has not been sufficiently taken into account, despite the existence of psychological support services in some hospitals. Possible avenues for improvement of psychological support to reinforcement workers without negatively affecting the well-being at work of regular ICU staff include the creation of a reserve pool of qualified staff. Future quantitative and qualitative studies are warranted to investigate the efficacy of such an approach.

## Supporting information

S1 Checklist (PDF)
